# Analysis of the miRNA Expression Profiles in the Zearalenone-Exposed TM3 Leydig Cell Line

**DOI:** 10.3390/ijms20030635

**Published:** 2019-02-01

**Authors:** Mingyang Wang, Weiwei Wu, Lin Li, Jianbin He, Sheng Huang, Si Chen, Jia Chen, Miao Long, Shuhua Yang, Peng Li

**Affiliations:** 1 Key Laboratory of Zoonosis of Liaoning Province, College of Animal Science & Veterinary Medicine, Shenyang Agricultural University, Shenyang 110866, China; m13940546231@163.com (M.W.); syndlilin@126.com (L.L.); hejianbin69@163.com (J.H.); used99@163.com (S.H.); 15541486321@163.com (S.C.); 15524373937@163.com (J.C.); 2 Institute of Animal Science, Xinjiang Academy of Animal Sciences, Urumqi 830000, China; wuweiweigp@foxmail.com; 3 Fushun modern agriculture and poverty alleviation and development promotion center, Fushun 113006, China

**Keywords:** zearalenone, microRNA, toxicology, TM3 Leydig cell, reproductive health

## Abstract

Zearalenone (ZEN), an important environmental pollutant, can cause serious harm to human and animal health. The aim of our study was to examine the effect of zearalenone (ZEN) on miRNA expression profiles in the mouse Leydig cell line (TM3 Leydig cell line) by miRNA sequencing. The effect of ZEN on the viability of TM3 Leydig cells was verified by Cell Counting Kit-8 (CCK-8). MiRNA sequencing was performed 24 h after the exposure of TM3 Leydig cells with 50 μmol/L of ZEN. Bioinformatics predicted the miRNA target genes, performed Gene ontology (GO) and Kyoto Encyclopedia of Genes and Genomes (KEGG) analyses, and conducted miRNA-gene-pathway mapping to show the relationship between miRNA, the target gene, and the signalling pathway. The expression levels of miRNA and the miRNA target genes associated with ZEN toxicology were verified by quantitative real-time polymerase chain reaction. The miRNA sequencing revealed a significant change (*p* < 0.05) in the 197 miRNAs in the ZEN-treated and control groups, among which 86 were up-regulated and 111 were down-regulated. GO analysis of the target genes of these miRNAs indicated various biological functions. KEGG analysis showed that the predicted miRNA target genes were involved in signalling pathways, such as cancer, apoptosis, and oxidation, namely, the Ras signalling pathway, Rap1 signalling pathway, PI3K-AKT signalling pathway, Foxo signalling pathway, and AMPK signalling pathway. These results suggest that ZEN, as an estrogen-like toxin, is regulated by microRNAs. Our results can help to examine the toxicological effects of ZEN-regulated miRNAs on germ cells.

## 1. Introduction

Zearalenone (ZEN) is a mycotoxin produced by *Fusarium fungi* [1,2]. The structure of ZEN is similar to that of 17β-oestradiol: ZEN competitively binds to estrogen receptors and activates the transcription of estrogen-responsive genes [3,4]. Therefore, ZEN plays a role by interfering with the physiological estrogen signalling pathway. ZEN may cause reproductive problems, such as ovarian dysfunction, decreased fertility, early abortion, reduced litter size, lower testicular weight, decreased motility of spermatozoa, and a lower total motile sperm count [5,6,7]. These reproductive toxicities are all related to the ZEN interference with the binding site of estrogen. However, some of the toxic effects of ZEN in an animal’s body cannot be explained simply by affecting the estrogen binding site. Some reports indicate that ZEN can cause oxidative stress and inflammation in animals. For example, studies showed that vitamin C could protect the liver of piglets by regulating the expression of nuclear receptors PXR and CAR and their target genes to prevent ZEN-induced oxidative stress [8]. Fan et al. demonstrated that ZEN-induced intestinal inflammation was mediated by NLRP3 and that ZEN could also affect cell apoptosis and autophagy by regulating target genes and signalling pathways [9]. These studies revealed that SIRT1 protects cardiac cells against apoptosis induced by ZEN or its metabolites α- and β-zearalenol through an autophagy-dependent pathway [10]. Long Miao found that procyanidins protect ZEN-induced apoptosis in mice through the Nrf2/ARE signalling pathway [11]. Therefore, the toxic effects of ZEN on animal organisms, such as oxidative stress, inflammatory response, apoptosis, and autophagy, need to be explained further by toxicological mechanisms.

MicroRNA (miRNA) is an 18–26 bp non-coding nucleotide sequence that affects the post-transcriptional gene expression by the specific base pairing of the 5’ (the seed) with the 3’ untranslated region of the target mRNA [12,13]; miRNAs are considered to act primarily by disrupting the cytoplasmic mRNA and regulating the mRNA translation (about 80%). A previous study reported that miRNAs could up-regulate the target mRNA during cell cycle arrest and inhibit translation in proliferating cells [14]. The miRNA maturation process involved in the nuclear processing of primary miRNA by DROSHA, nuclear export of precursor miRNA (pre- miRNA) by exportin 5, and cytoplasmic processing of pre- miRNA by DICER [15]. Recent studies showed that the differential expression of miRNAs in mouse Leydig cells was discovered by the addition of the brain-derived neurotrophic factor and luteinizing hormone during the cultivation of TM3 cells [16,17]. These studies show that miRNAs may be involved in the regulation of hormones in certain physiological functions of mouse Leydig cells. As a special type of estrogen, ZEN can compete with estrogen in vivo and cause reproductive damage to the body [3,4]. Whether the miRNAs after ZEN exposure to TM3 cells are involved in the regulation of germ cell toxicology is unclear.

Clinical studies should determine whether and how miRNAs participate in the toxicological processes of germ cells by miRNA sequencing. Therefore, this study provides a theoretical basis for the molecular toxicological studies of ZEN. At present, ZEA has been thoroughly explained to have many toxic effects at the mRNA level, but whether miRNA is involved in the toxicological effects of ZEA and the mechanism of the toxicological action of miRNA in ZEA have not been elucidated. Only a relatively few studies have been conducted on these issues, and further research is needed. Thus, on the basis of the ZEA-infected cell model, we searched for differentially expressed miRNAs and combined them with biological information technology to analyze the relationship between differentially expressed miRNAs and ZEA-induced cytotoxicity. We aimed to provide a theoretical basis for future molecular studies on the toxicology of ZEN.

## 2. Results

### 2.1. Effect of ZEN on the Proliferation of TM3 Leydig Cells

**Figure 1:**
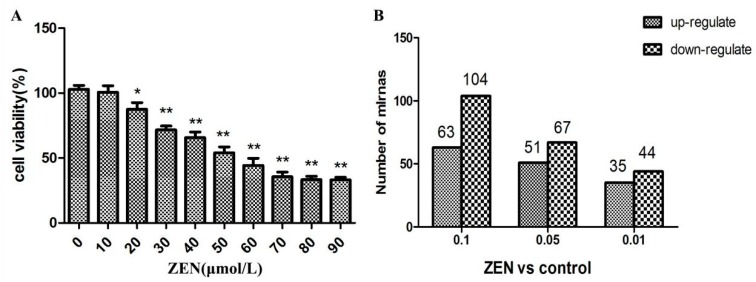
(**A**) Effect of zearalenone (ZEN) on the proliferation of TM3 Leydig cells. Cytotoxic effects of ZEN on TM3 Leydig cells. Cells were treated with increasing concentrations (0–90 μmol/L) of ZEN for 24 h and then processed for the CCK-8 assay. Data are expressed as the mean ± S.E.M. of three independent experiments. * *p* < 0.05; ** *p* < 0.01. (**B**) Differential expression of miRNA in the ZEN exposure groups.

After 24 h of incubation, ZEN treatment markedly inhibited the cell viability in a dose-dependent manner (Figure 1A).

### 2.2. Differential Expression of miRNAs in the ZEN Exposure Groups

The miRNA deep sequencing analysis was used to identify the differential expression of miRNAs in the TM3 Leydig cells between the ZEN-treated and control groups. In the miRNA sequencing results, 197 miRNAs changed significantly (fold change > 1.5, *p* < 0.05), with 86 miRNAs up-regulated and 111 miRNAs down-regulated (Figure 1B).

### 2.3. Prediction and Functional Classification of Target Genes

**Figure 2:**
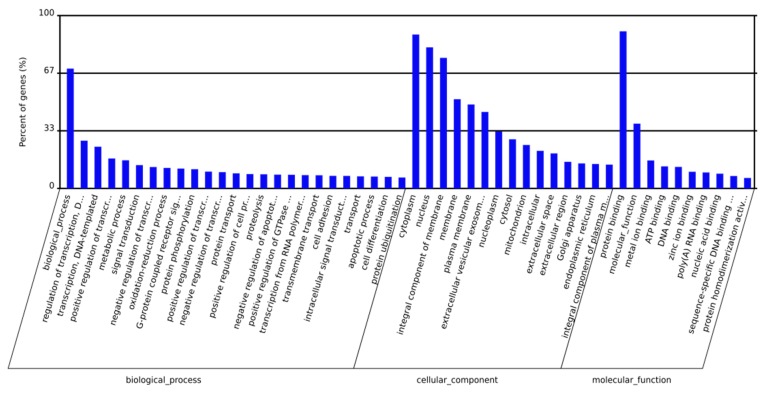
**Gene ontology** (GO) term enrichment of the predicted targets of differentially expressed miRNAs. GO term enrichment showed that the putative targets of the differentially expressed miRNAs were associated with diverse functional terms, including binding, membrane, and reproduction.

For the target animal species, Target Gene and MiRanda software were used to perform target gene predictions for miRNAs with significant differences. The predicted genes were classified into 3 Gene ontology (GO) categories and 50 terms were enriched from gene ontology analysis (Figure 2). Twenty-five terms were enriched to the biological process category, including “biological process” and “regulation of transcription”. Fifteen terms were enriched to the cellular component category, including “cytoplasm”, “nucleus”, and “integral components of membrane”. Ten terms were enriched to the molecular function category, including “protein binding”, “molecular function”, and “metal ion binding”.

**Figure 3:**
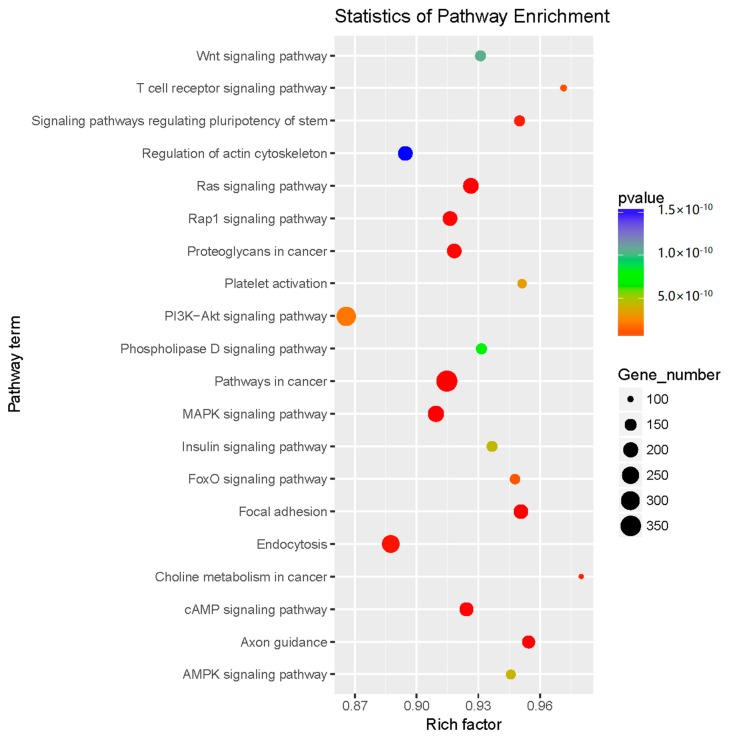
Pathway enrichment of the predicted targets of the differentially expressed miRNAs. KEGG pathway analysis showed that the predicted targets were involved in cancer, apoptosis, and signalling pathways, such as the rat sarcoma signalling pathway (Ras signalling pathway), Ras-related protein1 signalling pathway (Rap1 signalling pathway), Phosphatidylinositide 3-kinases/AKT signalling pathway (PI3K-AKT signalling pathway), Forkhead boxo signalling pathway (Foxo signalling pathway), Adenosine 5‘-monophosphate (AMP)-activated protein kinase (AMPK signalling pathway), and mitogen-activated protein kinase signalling pathway (MAPK signalling pathway).

Pathway significance enrichment analysis revealed that the ZEN-affected miRNA target genes have a close relationship with signal pathways, such as the Ras signalling pathway, the pathway in cancer, Foxo signalling pathway, and endocytosis. The number of miRNA target genes in these pathways is significant. These pathways have a regulatory effect on germ cell apoptosis, autophagy, oxidative stress, cancer development, invasion, and differentiation. The above signal pathways were the focus of the research on the effects of ZEN on germ cells (Figure 3). Some miRNAs likely to be associated with ZEN toxicology were screened by GO and Kyoto Encyclopedia of Genes and Genomes (KEGG) enrichment analysis. The results are shown in Table 1.

**Figure 4:**
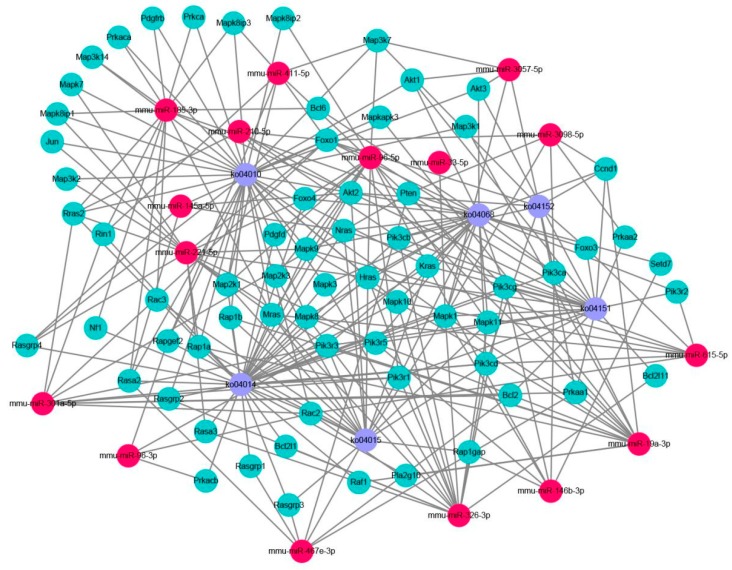
The miRNA-gene-pathway interactive map. Red indicates miRNA, light green indicates target gene, and purple indicates signal pathway. The signal path uses the following ko codes: ko04014 (Ras signalling pathway), ko04015 (Rap1 signalling pathway), ko04151 (PI3K-AKT signalling pathway), ko04068 (Foxo signalling pathway), ko04152 (AMPK signalling pathway), and ko04010 (MAPK signalling pathway).

We mapped the miRNA-gene-pathway interaction map based on the GO and KEGG analyses (Figure 4). One miRNA can regulate multiple target genes, and one target gene is regulated by multiple miRNAs. The target genes regulated by miRNAs regulate the corresponding pathways. Through the miRNA-gene-pathway interaction map, the relationship between the miRNA, target genes, and signalling pathways can be clearly seen. However, these signalling pathways are closely related to the toxicological mechanisms of ZEN.

### 2.4. Validation of the Differentially Expressed miRNAs

**Figure 5:**
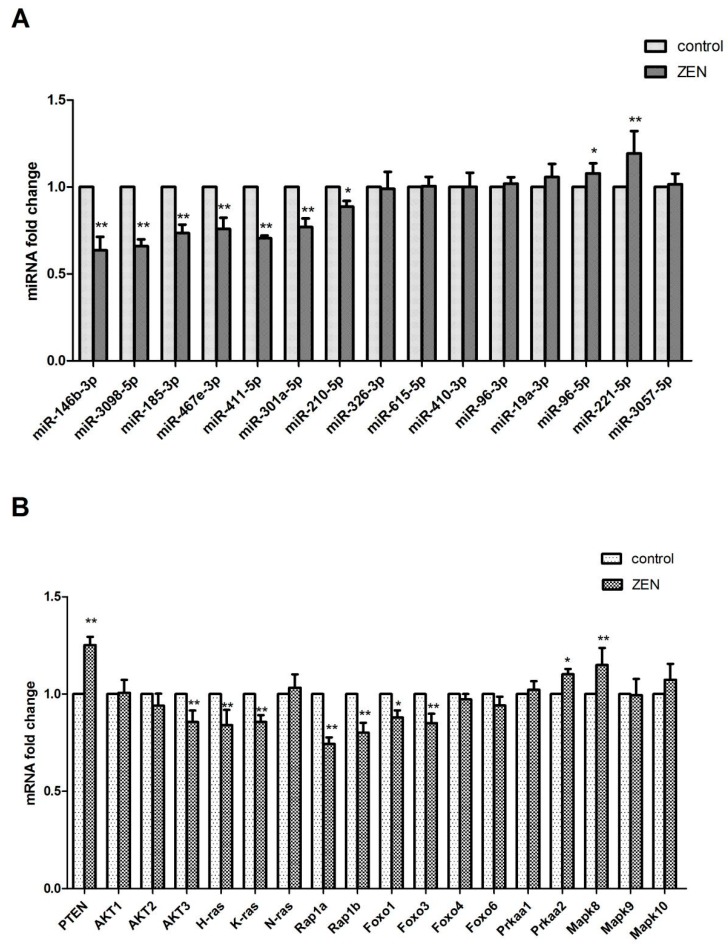
(**A**) Validation of the differentially expressed miRNAs. (**B**) Verification of the miRNA target genes. The qRT-PCR analyses of the mRNA expression levels ofmiR-146b-3p, miR-3098-5p, miR-185-3p, miR-467e-3p, miR-411-5p, miR-301a-5p, miR-210-5p, miR-326-3p, miR-615-5p, miR-410-3p, miR-96-3p, miR-19a-3p, miR-96-5p, miR-221-5p, miR-3057-5p, PTEN, AKT1, AKT2, AKT3, H-ras, N-ras, K-ras, Rap1a, Rap1b, Foxo1, Foxo3, Foxo4, and Foxo6 in the control and ZEA-exposed TM3 Leydig cells. The gene expression levels represent the mRNA expression levels relative to the control levels (the values represent mean ± SD). The asterisks are used to indicate a statistically significant difference: * *p* < 0.05; ** *p* < 0.01.

From the references and miRNA targets, 16 differentially expressed miRNAs, namely, miR-146b-3p, miR-3098-5p, miR-185-5p, miR-467e-3p, miR-441-5p, miR-301a-5p, miR-210-5p, miR-195a-3p, miR-326-3p, miR-615-3p, miR-410-3p, miR-96-3p, miR-96-5p, miR-467e-3p, miR-19a-3p, and miR-221-5p, were selected for qRT-PCR analysis. Compared with those in the control group, miR-96-5p, miR-467e-3p, miR-19a-3p and miR-221-5p exhibited a significantly increased expression. By contrast, miR-146b-3p, miR-3098-5p, miR-185-5p, miR-467e-3p, miR-441-5p, miR-301a-5p, miR-210-5p, miR-195a-3p, miR-326-3p, miR-615-3p, miR-410-3p, and miR-96-3p showed a significantly decreased expression in the ZEN-treated group (*p* < 0.05) (Figure 5A). These results showed that the expression levels of all 16 miRNAs analyzed by qRT-PCR were in accordance with the deep sequencing data.

### 2.5. Verification of the miRNA Target Genes

As shown in Figure 5B, we verified the expression levels of the miRNA target genes in the Ras signalling pathway, Rap1 signalling pathway, PI3K-AKT signalling pathway, Foxo signalling pathway, AMPK signalling pathway, and MAPK signalling pathway by qRT-PCR. The findings are consistent with the sequencing results.

## 3. Discussion

The mechanism of ZEN toxicity to humans and animals is complicated. Elucidating the mechanism of ZEN toxicity to humans and animals is of great clinical significance to prevent and treat ZEN. Recent studies have shown that the addition of reproductive hormones to TM3 cells affects the expression of miRNAs in TM3 Leydig cells [17]. Therefore, we speculate that miRNAs are involved in the regulation of ZEN toxicology after ZEN is exposed to TM3 cells.

GO enrichment analyses were performed for the miRNA target genes. The results showed that a lot of GO terms were enriched. Specifically, the GO term “cytoplasm”, “nucleus”, “integral component of membrane”, and “protein binding” were the most significantly enriched. Coincidently, ZEN can affects the cellular structure and cellular connections of germ cells [18]. Therefore, we hypothesized a correlation between the toxicological effects of ZEN and these GO terms.

We selected some signalling pathway related to ZEN toxicology through GO and KEGG analyses. The Ras signalling pathway, Rap1 signalling pathway, PI3K-AKT signalling pathway, Foxo signalling pathway, AMPK signalling pathway, MAPK signalling pathway, and other signalling pathways were used to map the miRNA-gene-pathway interaction. The miRNA-gene-pathway interaction map clearly showed the mutual regulation among these six signalling pathways. As indicated by the KEGG analysis, miRNA could be involved in the regulation of target genes in these six signalling pathways by ZEN and affect the cell phenotype of germ cells.

Ras oncoprotein plays a key role in the development and maintenance of many tumor types [19,20]. Rap1 and Ras have a high degree of sequence similarity, have overlapping binding partners and have been shown to antagonize and mimic the Ras-driven cancer phenotype [21]. Studies have shown that ZEN, as a natural female-like toxin, mainly produces carcinogenic effects by interfering with endocrine balance [22]. In our results, the results of the KEGG analysis were significant in the Ras and Rap1 signalling pathways; the expressions of H-ras, K-ras, Rap1a, and Rap1b genes in the Ras and Rap1 proteins were significantly regulated, and H-ras, K-ras, Rap1a, and Rap1b were regulated. The expression of the isogenic miRNA (Figure 4 and Figure 5) was also significant. This finding suggests that miRNAs may be involved in the toxicological process of ZEN carcinogenesis through the Ras and Rap1 signalling pathways. In addition, some scholars reported that ZEN influences the tumorigenic mechanism of TM3 Leydig cells by affecting proto-oncogenes [23]. Our research provides a new idea for the tumorigenic mechanism of ZEN.

The PI3K-AKT signalling pathway is an anti-apoptotic pathway that regulates the expression of downstream target proteins, such as Bax and Bcl-2, and thus participates in the regulation of cell growth, apoptosis, differentiation, migration, invasion, and angiogenesis [24,25]. The PTEN protein upstream of the PI3K-AKT signalling pathway can dephosphorylate phosphatidylinositol triphosphate to phosphatidylinositol diphosphate, thereby negatively regulating the PI3K-AKT signalling pathway and playing an important role in apoptosis [26,27]. The Foxo signalling pathway is located downstream of the PI3K-AKT signalling pathway and is regulated by the PI3K-AKT signalling pathway. The Foxo signalling pathway is closely related to apoptosis and oxidative stress [28,29]. A large number of studies have shown that ZEN can produce toxicological effects, such as oxidative stress and apoptosis, on germ cells. Long et al. investigated the oxidative damage of testis induced by ZEN in male mice and the apoptosis of ZEN induced by the Nrf2/ARE signalling pathway [11,30]. ZEN is known to cause changes in cell phenotypes, such as oxidative stress and apoptosis, in germ cells through the signalling pathways. In our experiments, genes such as PTEN, AKT2, AKT3, Foxo1, Foxo3, and Foxo6 were significantly expressed in the PI3K-AKT signalling pathway and the Foxo signalling pathway. The expression of miRNAs (Figure 4 and Figure 5) regulating PTEN, AKT2, AKT3, Foxo1, Foxo3, and other genes was significant. This finding suggests that ZEN may cause germ cell apoptosis through the PI3K-AKT signalling pathway and the Foxo signalling pathway, and that miRNA may participate in the regulation of germ cell apoptosis through ZEN by affecting the target genes in the PI3K-AKT signalling pathway and the Foxo signalling pathway. Although no studies have shown that ZEN induces germ cell apoptosis through the PI3K-AKT signalling pathway and the Foxo signalling pathway, our study provides strong evidence of ZEN being able to induce germ cell apoptosis through the PI3K-AKT signalling pathway and the Foxo signalling pathway.

In the AMPK signalling pathway, the expression of the *Prkaa2* genes that regulate the AMPK protein is significant, and the differential expression of the miRNAs that regulate the *Prkaa2* genes (Figure 4 and Figure 5) is also significant. Currently, studies have shown that in addition to the classical pathway of endoplasmic reticulum-induced apoptosis, the ATP/AMPK signalling pathway is regulated by endoplasmic reticulum stress during apoptosis induced by ZEN [31]. Therefore, through our experimental results, we boldly speculate that miRNA may be involved in the regulation. In addition, Pistol et al. [32] verified by transcriptome sequencing that the ZEN inflammatory stimuli and immunotoxicity in pig spleen cells could be the result of the JNK pathway activation but not that of the p-38/MAPK and NF-kB genes and proteins. On the basis of their results, we validated the JNK and JUN genes Mapk-8 and Mapk-10 as well as the JUN genes and their miRNAs (Figure 4 and Figure 5) in the MAPK signalling pathway, and the results were all significant. This outcome suggests that the inflammatory stimuli and immunotoxicity produced by the ZEN activation of the JNK pathway may involve miRNAs. Our research provides a theoretical basis for this assumption.

Our results of this study reveal a complex network of miRNA associated with diverse molecular functions, which may be engaged in cellular response to ZEA exposure. However, more detailed aspects of these findings should be elucidated in future research, such as luciferase assay or gene knockout experiment, for better characterization of genes selected in this study.

## 4. Materials and Method

### 4.1. Cell Culture and ZEN Exposure

Mouse TM3 Leydig cells were obtained from the American Type Culture Collection (Beijing, China). The cells were seeded as monolayer cultures in Dulbecco’s modified Eagle ‘s medium/F-12 (HyClone, Logan, UT, USA) with 10% (*v*/*v*) inactivated fetal bovine serum and 1% (*v*/*v*) penicillin/streptomycin incubated at 37 °C with 5% CO_2_. When the volume of adherent cells occupied 80% of the dish, the cells were treated with 50 μmol/L of ZEN for 24 h. Each treatment was replicated thrice.

### 4.2. Cell Viability Assay

The cytotoxic effects of ZEN on TM3 cells were determined using a Cell Counting Kit-8 (CCK-8) (Solarbio, Beijing, China) assay. Cells were plated at a density of 2 × 10^5^ per well in 96-well plates. After treatment with 0–90 μmol/L ZEN for 24 h, 10 μL of CCK-8 was added to each well, and the cells were incubated for 2 h at 37 °C. Non-treated cells served as a negative control. The absorbance was determined at a wavelength of 450 nm using a microplate reader (Infinite 200 PRO, ABI, New York, NY, USA). The results are presented as percentage of the values measured for untreated control cells. It has been reported that the IC_50_ of ZEN-treated TM3 cells is 50 μmol/L [33].

### 4.3. Small RNA Sequencing and Bioinformatics Analysis

Total RNA was extracted using Trizol reagent (Invitrogen, Carlsbad, CA, USA) following the manufacturer’s procedure. The total RNA quantity and purity were analyzed by Bioanalyzer 2100 and RNA 6000 Nano LabChip Kit (Agilent, Palo Alto, CA, USA) with RIN number greater than 7.0. Approximately 1 µg of the total RNA was used to prepare a small RNA library according to the protocol of TruSeq Small RNA Sample Prep Kits (Illumina, San Diego, CA, USA). We performed single-end sequencing (36 bp) on an Illumina Hiseq2500 in the Lianchuan Biotechnology (Hangzhou, China) following the vendor’s recommended protocol.

Raw reads were subjected to an in-house program, ACGT101-miR (LC Sciences, Houston, TX, USA) to remove adapter dimers, junk, low complexity, common RNA families (rRNA, tRNA, snRNA, snoRNA), and repeats. Subsequently, unique sequences with length in 18~26 nucleotide were mapped to specific species precursors in miRBase 21.0 (http://www.mirbase.org/) by BLAST search to identify known miRNAs and novel 3p- and 5p- derived miRNAs. Length variation at both 3′ and 5′ ends and one mismatch inside of the sequence were allowed in the alignment. The unique sequences mapping to specific species mature miRNAs in hairpin arms were identified as known miRNAs. The unique sequences mapping to the other arm of the known specific species precursor hairpin opposite to the annotated mature miRNA-containing arm were considered to be novel 5p- or 3p- derived miRNA candidates. The remaining sequences were mapped to other selected species precursors (with the exclusion of specific species) in miRBase 21.0 by BLAST search, and the mapped pre-miRNAs were further BLASTed against the specific species genomes to determine their genomic locations. The above two were defined as known miRNAs. The unmapped sequences were BLASTed against the specific genomes, and the hairpin RNA structures containing sequences were predicated from the flank 80 nt sequences using RNAfold software (http://rna.tbi.univie.ac. at/cgi-bin/RNAfold.cgi).

To predict the genes targeted by the differentially expressed miRNAs, two computational target prediction algorithms (TargetScan 50 and miRanda 3.3a) were used to identify the miRNA binding sites. The data predicted by both algorithms were then combined, and the overlaps were calculated. The GO terms and KEGG pathway of these differentially expressed miRNA targets were also annotated. Then, we mapped the predicted target genes to map the miRNA-gene-pathway interactions.

### 4.4. Quantitative Real-Time Polymerase Chain Reaction (qRT-PCR)

Total RNA was extracted using Trizol reagent (Invitrogen, Carlsbad, CA, USA) following the manufacturer’s procedure. The total RNAs from each sample were reverse-transcribed to cDNA using the miRNA first-strand cDNA synthesis kit (by stem-loop) (Vazyme, Nanjing, China). The qRT- PCR was performed using the miRNA Universal SYBR qPCR Master Mix (Vazyme, Nanjing, China). Reverse transcription reactions were performed at the following parameters: 25 °C (mRNA) for 5 min, 50 °C for 15 min, and 85 °C for 5 min. PCR reactions were performed at the following parameters: 95 °C for 5 min followed by 40 cycles of 95 °C for 10 s and 60 °C for 30 s. A U6 small nuclear RNA was used as the endogenous control for the data normalization of miRNAs, and β-actin was then adopted as the internal control of mRNA expression. The relative expression was calculated by the comparative threshold cycle method. The sequences of the primers used for reverse transcription and qRT-PCR were purchased from Sangon Biotech Company, Shanghai, China (Table 2 and Table 3).

### 4.5. Statistical Analysis

The experiments of cell growth analysis and qRT-PCR were performed with three technical replicates. All values are presented as mean ± SEM. For the small RNA sequencing data, the threshold values we used in choosing the differentially expressed miRNAs were a fold change greater than 1.5 and a *p* value less than 0.05. For the qRT-PCR data, statistical analysis was performed using the Statistical Package for Social Sciences version 22.0 (IBM, Armonk, NY, USA). The fold changes were calculated through the relative quantification with 2^−ΔΔCT^. A *p* value less than 0.05 was considered significant.

## 5. Conclusions

In summary, differentially expressed miRNAs in TM3 Leydig cells after ZEN exposure were identified. Sixteen miRNAs and target genes were identified to exhibit differential expression. GO enrichment analysis and pathway interaction analysis showed that miRNA and target genes are closely related to the toxicological mechanism of ZEN. QRT-PCR analysis of representative genes indicated that Ras signalling pathway, Rap1 signalling pathway, PI3K-AKT signalling pathway, Foxo signalling pathway, AMPK signalling pathway, and MAPK signalling pathway lead to oxidative stress, apoptosis, and carcinogenesis of germ cells. This study provides a molecular basis and new insights into the toxicological mechanisms of ZEN.

## Figures and Tables

**Table 1:** The miRNAs associated with ZEN toxicology.

miRNA Name	log2 (Fold Change) ^a^	*p* Value (Chi_Square_2×2)	*p* Value (Fisher Test)
miR-146b-3p	−2.5	3.01 × 10^−3^	9.29 × 10^−3^
miR-3098-5p	−2.26	8.57 × 10^−3^	3.38 × 10^−2^
miR-185-3p	−2.12	5.42 × 10^−4^	1.11 × 10^−3^
miR-467e-3p	−2.09	3.24 × 10^−3^	2.43 × 10^−3^
miR-411-5p	−1.95	6.82 × 10^−18^	1.09 × 10^−17^
miR-301a-5p	−1.71	2.14 × 10^−6^	3.17 × 10^−6^
miR-210-5p	−1.61	1.23 × 10^−10^	3.57 × 10^−10^
miR-326-3p	−1.30	2.59 × 10^−4^	6.30 × 10^−4^
miR-615-5p	−1.23	9.62 × 10^−51^	2.25 × 10^−49^
miR-410-3p	−1.17	8.96 × 10^−4^	1.79 × 10^−3^
miR-96-3p	−1.06	5.13 × 10^−3^	1.04 × 10^−2^
miR-19a-3p	1.21	1.02 × 10^−7^	6.96 × 10^−8^
miR-96-5p	1.47	3.67 × 10^−47^	1.28 × 10^−49^
miR-221-5p	2.0	1.63 × 10^−62^	2.13 × 10^−68^
miR-3057-5p	2.01	4.49 × 10^−11^	8.80 × 10^−12^

Note: ^a^ log2 (fold change) refers to the relative expression levels between ZEN-exposed to control groups generated by sequencing data. Positive Expressions Up and Negative Expressions Down.

**Table 2:** Primer sequences of mRNA used in this study for quantitative real-time RT-PCR.

Gene	Primer Sequence (5′-3′)	Accession No
β-actin	Forward: CTGTCCCTGTATGCCTCTG Reverse: TTGATGTCACGCACGATT	BC_138614.1
N-ras	Forward: GGTTGGAGCAGGTGGTGTT Reverse: TTTCGGTAAGAATCCTCTATG	NM_010937.2
K-ras	Forward: TGCCTTCTAGAACAGTAGACAC Reverse: CTTTGCTGAGGTCTCAATGAAC	NM_021284.6
H-ras	Forward: GCATCCCCTACATTGAAACATC Reverse: CAATTTATGCTGCCGAATCTCA	NM_001130443.1
Pten	Forward: TGGATTCGACTTAGACTTGACC Reverse: TCACTTAGCCATTGGTCAAGAT	NM_008960.2
Akt1	Forward: TGCACAAACGAGGGGAATATAT Reverse: CGTTCCTTGTAGCCAATAAAGG	NM_001165894.1
Akt2	Forward: TCGATTATCTCAAACTCCTCGG Reverse: CGACTTCATCCTTTGCAATGAT	NM_001110208.2
Akt3	Forward: GGGGTGGAACAGTAAAGACA Reverse: GCATTATGAGCAGTGGAGG	NM_011785.4
Rap1a	Forward: ATTCCTACAGAAAGCAAGTCGA Reverse: ATCTTCTGTGTCTTTAACCCGT	NM_145541.5
Rap1b	Forward: AAGCAAGTTGAAGTAGATGCAC Reverse: CATCATCAGTGTCTTTAACCCG	NM_024457.2
Foxo1	Forward: GATCTACGAGTGGATGGTGAAG Reverse: GACAGATTGTGGCGAATTGAAT	NM_019739.3
Foxo3	Forward: TCACTGTATTCAGCTAGTGCAA Reverse: ATGATGGACTCCATGTCACATT	NM_019740.2
Foxo4	Forward: GAATCCTGGGGGCTGTAAC Reverse: GCTGATGAGTTCTGCATATGAC	NM_018789.2
Foxo6	Forward: GAAAGCGAAGAGCTCCCGAC Reverse: GTGCCGAATGGAGTTCTTCCAG	NM_194060.1
Prkaa1	Forward: GGACTTACTTGTTGGATTTCCG Reverse: CCTTTGGCAAGATCGATAGTTG	NM_001013367.3
Prkaa2	Forward: GTGGTGACCCTCAAGACCAG Reverse: GTGGTTTCAAGCCTGGAGGA	NM_001356568.1
Mapk8	Forward: TTGAAAACAGGCCTAAATACGC Reverse: GTTTGTTATGCTCTGAGTCAGC	NM_001310452.1
Mapk9	Forward: GTGGAAAACAGACCAAAGTACC Reverse: CATGCTCTCTTTCTTCCAACTG	NM_001163671.1
Mapk10	Forward: CACGAGCGGATGTCTTACT Reverse: TTGACTACAATGTTACTGGGTT	NM_001081567.2

**Table 3:** Primer sequences of miRNA used in this study for quantitative real-time RT-PCR.

miRNA	Primer sequence (5′-3′)
U6	RT: CGCTTCACGAATTTGCGTGTCAT Forward: GCTTCGGCAGCACATATACTAAAAT Reverse: CGCTTCACGAATTTGCGTGTCAT
miR-146b-3p	RT: GTCGTATCCAGTGCAGGGTCCGAGGTATTCGCACTGGATACGACACCAGA Forward: GCGGCCCTAGGGACTCAGT Reverse: AGTGCAGGGTCCGAGGTATT
miR-185-3p	RT: GTCGTATCCAGTGCAGGGTCCGAGGTATTCGCACTGGATACGACACCAGA Forward: CGAGGGGCTGGCTTTCC Reverse: AGTGCAGGGTCCGAGGTATT
miR-3098-5p	RT: TGACCGTCTGTATGGTTGTTCACGACTCCTTCACCCTATCCAACCATACAGACGGTCAGCTCCTAC Forward: GGGTCCTAACAGCAGGAGTA Reverse: TATGGTTGTTCACGACTCCTTCAC
miR-467e-3p	RT: TGACCGTCTGTATGGTTGTTCACGACTCCTTCACCCTATCCAACCATACAGACGGTCAATATAGGTG Forward: GGGATATACATACACACAC Reverse: TATGGTTGTTCACGACTCCTTCAC
miR-411-5p	RT: TGACCGTCTGTATGGTTGTTCACGACTCCTTCACCCTATCCAACCATACAGACGGTCACGTACGCT Forward: GGGTAGTAGACCGTATAGC Reverse: TATGGTTGTTCACGACTCCTTCAC
miR-326-3p	RT: TGACCGTCTGTATGGTTGTTCACGACTCCTTCACCCTATCCAACCATACAGACGGTCAACTGGAGG Forward: GGGCCTCTGGGCCCTTCCT Reverse: TATGGTTGTTCACGACTCCTTCAC
miR-615-5p	RT: TGACCGTCTGTATGGTTGTTCACGACTCCTTCACCCTATCCAACCATACAGACGGTCAGATCCGAG Forward: GGGGGTCCCCGGTGCT Reverse: TATGGTTGTTCACGACTCCTTCAC
miR-410-3p	RT: TGACCGTCTGTATGGTTGTTCACGACTCCTTCACCCTATCCAACCATACAGACGGTCAACAGGCCA Forward: GGGAATATAACACAGATGG Reverse: TATGGTTGTTCACGACTCCTTCAC
miR-96-3p	RT: TGACCGTCTGTATGGTTGTTCACGACTCCTTCACCCTATCCAACCATACAGACGGTCAATATTGGC Forward: GGGCAATCATGTGTAGTGC Reverse: TATGGTTGTTCACGACTCCTTCAC
miR-19a-3p	RT: TGACCGTCTGTATGGTTGTTCACGACTCCTTCACCCTATCCAACCATACAGACGGTCATCAGTTTTG Forward: GGGTGTGCAAATCTATGCAA Reverse: TATGGTTGTTCACGACTCCTTCAC
miR-3057-5p	RT: TGACCGTCTGTATGGTTGTTCACGACTCCTTCACCCTATCCAACCATACAGACGGTCAATCCCGCA Forward: GGGATTGGAGCTGAGATTCTG Reverse: TATGGTTGTTCACGACTCCTTCAC
miR-301a-5p	RT: GTCGTATCCAGTGCAGGGTCCGAGGTATTCGCACTGGATACGACAGTAGT Forward: CGCGGCTCTGACTTTATTGC Reverse: AGTGCAGGGTCCGAGGTATT
miR-221-5p	RT: GAGGTATTCGCACTGGATACGACACAGAA Forward: GCGACCTGGCATACAATGTAGAT Reverse: AGTGCAGGGTCCGAGGTATT
miR-96-5p	RT: GTCGTATCCAGTGCAGGGTCCGAGGTATTCGCACTGGATACGACAGCAAA Forward: GCGTTTGGCACTAGCACATT Reverse: AGTGCAGGGTCCGAGGTATT
miR-210-5p	RT: TGACCGTCTGTATGGTTGTTCACGACTCCTTCACCCTATCCAACCATACAGACGGTCACAGTGTGC Forward: GGGAGCCACTGCCCACCGC Reverse: TATGGTTGTTCACGACTCCTTCAC
